# Prognostic impact of lymphovascular invasion in node-negative gastric cancer: a retrospective cohort study

**DOI:** 10.1186/s12957-024-03629-6

**Published:** 2024-12-20

**Authors:** Abdullah Ibrahim Alangari, Sojung Kim, Han Hong Lee, Kyo Young Song, Hoseok Seo

**Affiliations:** 1 Division of Gastrointestinal Surgery, Department of Surgery, Seoul St. Mary’s Hospital, College of Medicine, The Catholic University of Korea, 222, Banpo-daero, Seocho-gu, Seoul, 06591 Republic of Korea; 2 Department of Surgery, Al Nakeel Medical Center, Ministry of Defense, Riyadh, Saudi Arabia

**Keywords:** Stomach neoplasms, Lymphovascular invasion, Prognosis, Gastrectomy, lymphatic metastasis

## Abstract

**Background:**

Lymphovascular invasion (LVI) has been identified as a prognostic factor in various cancers, but its significance in node-negative gastric cancer remains unclear. Gastric cancer prognosis is notably affected by lymph node metastasis, with LVI potentially indicating metastatic spread.

**Methods:**

A retrospective review was conducted on 5,699 patients who underwent curative radical gastrectomy for gastric cancer between 1989 and 2018. The median follow-up duration was 62 months (0–362 months). Overall, disease-specific, and disease-free survival were compared based on LVI status and stratified by T stage. Additionally, patients with stage IIA or T2N0 were further evaluated to clarify the clinical significance of LVI in the T2N0 group.

**Results:**

The T2N0 LVI-positive group exhibited significantly poor prognosis than those in the T2N0 LVI-negative group, with no significant differences observed on comparing the T2N0 LVI-positive group with the T2N1 LVI-negative or LVI-positive groups. Furthermore, although the T2N0 LVI-negative group demonstrated better prognosis compared to the IIA group, the T2N0 LVI-positive group exhibited worse survival. In addition, LVI positivity was an independent risk factor for overall survival in T2N0 patients.

**Conclusions:**

LVI in node-negative gastric cancer has clinical significance as a prognostic indicator, indicating an increased risk of disease recurrence and poor survival especially in T2 cohort. This indicates an increased likelihood of lymph node involvement and may influence treatment decisions and follow-up strategies.

**Supplementary Information:**

The online version contains supplementary material available at 10.1186/s12957-024-03629-6.

## Introduction

Gastric cancer remains a significant global health challenge, ranking as one of the leading causes of cancer-related mortality worldwide [1]. The prognosis of gastric cancer is closely associated with the presence of lymph node (LN)metastasis. In cases of advanced gastric cancer (T2 or higher), the presence of LN metastasis serves as an indication for adjuvant chemotherapy [2]. This is due to the significantly poorer outcomes observed in patients with LN positive gastric cancer compared to those without nodal involvement [3, 4]. 

Risk factors for LN metastasis in gastric cancer include tumor size, depth of invasion, histologic type, and presence of lymphovascular invasion (LVI) [5, 6]. Notably, the criteria for endoscopic submucosal dissection (ESD) also consider these factors to minimize the risk of overlooking potential LN metastasis [7]. The accuracy of pathologic reports on LN status could be influenced by the extent of LN retrieval and the anatomical section examined. Consequently, a diagnosis of node-negative gastric cancer might not preclude the presence of undetected LN metastasis, potentially leading to a false-negative assessment and a poor prognosis [8, 9]. 

LVI, which is characterized by the invasion of vessel walls by tumor cells and/or the presence of tumor emboli inside an endothelial-lined area, are the earliest indicator of LN metastasis or distant metastasis [10]. The prevalence and predictive value of LVI vary significantly across different types of cancer, including colorectal cancer, urothelial carcinoma, and breast cancer, where it is recognized as a prognostic factor [11–14]. 

This study aims to elucidate the clinical significance of LVI in patients with node-negative gastric cancer, particularly its role in predicting the likelihood of LN metastasis. By understanding the implications of LVI, we could better stratify patients for appropriate their therapeutic interventions and improve prognostic assessments in this patient population.

## Materials and methods

### Patient population and data collection

Patients diagnosed with gastric adenocarcinoma who underwent curative radical gastrectomy between 1989 and 2018 at Seoul St. Mary’s Hospital were considered for inclusion. The criteria for inclusion were pathologically confirmed primary gastric adenocarcinoma, R0 resection (indicating no macroscopic or microscopic tumor remnants), and complete data availability. Exclusions were made for patients with metastatic lesions, those who received preoperative chemotherapy or radiation therapy, and those lacking information on LVI status. After applying these criteria, 5,699 patients were included in the study. Data on demographics, clinical and pathologic characteristics, operative details, long-term recurrence, and survival were collected retrospectively.

Preoperative clinical characteristics were categorized based on the Eastern Cooperative Oncology Group (ECOG) performance status [15]. Surgical procedures adhered to the Korean Gastric Cancer Treatment Guidelines [2]. Pathologic staging was determined using the 8th edition of the American Joint Cancer Committee TNM classification system [16]. LN sorting was performed using a combination of fine LN sorting and regional LN sorting methods [17]. The surgeon separated lymph nodes by station from the stomach on the operating room bench and delivered them to the pathology department. The pathologist then picked the lymph nodes from each station and prepared pathology slides. The pathological criteria for determining LVI were based on the guidelines of pathologic report for gastric cancer [10]. 

Patients were followed up every 3 or 6 months for the first 5 years post-surgery and then annually until death or loss to follow-up. The median follow-up duration was 62 months (range: 0 to 362 months). Adjuvant chemotherapy was administered based on the stage, overall health, and patient preference. Indications for adjuvant chemotherapy were a pathologic stage of IIA or higher. The primary chemotherapy regimens were fluoropyrimidine-based or platinum-combination therapies, with the addition of irinotecan or taxane on a case-by-case basis. Locoregional recurrence was defined as recurrence in the remnant stomach, anastomosis site, or perigastric LNs. Distant LN recurrence was identified as recurrence in distant LNs or the ovaries. Distant organ recurrence was defined as recurrence in other organs, such as the lungs, liver, or bones, and peritoneal recurrence was defined as recurrence within the peritoneal cavity. In cases where recurrence involved multiple sites, classification was based on the site with the highest severity.

This study received approval from the institutional review board of the ethics committee of the College of Medicine at the Catholic University of Korea (approval no. KC23RISI0702). All patient records were anonymized and de-identified before analysis.

Cohorts.

Patients were grouped into five cohorts based on pathologic stage: (1) T1 cohort, (2) T2 cohort, (3) T3 cohort, (4) T4 cohort, and (5) TNM stage IB and IIA cohort. The T1 to T4 cohorts were further divided into four groups based on N stage and LVI status. Groups A and B were N0, and groups C and D were N1. The IB and IIA cohort was divided into four groups based on pathologic stage: (1) IIA, (2) T2N0 LVI positive, (3) T2N0 LVI negative, and (4) T1N1. Groups 2 to 4 were in the pathologic stage IB. The definitions of the cohorts and groups are detailed in Supplemental Table 1.

### Statistical analysis

Categorical variables were analyzed using the chi-square or Fisher’s exact test, as appropriate. Continuous variables are presented as means ± standard deviations and were compared using the Student’s t-test or an analysis of variance (ANOVA). Survival rates were analyzed using Kaplan-Meier survival curves. A Cox regression analysis was performed to identify the risk factors for survival. A p-value of < 0.05 was considered statistically significant. All statistical evaluations were conducted using SPSS (version 24; SPSS, Inc., Chicago, IL, USA) for Windows.

## Result

### Patient demographic and clinicopathological characteristics

A total of 5,699 patients were included. The clinicopathologic characteristics based on LVI presence are detailed in Supplemental Table 2. The LVI positive group had older patients, more open surgical approaches, and more total gastrectomies. There were also more cases with D2 or more extensive dissections. Tumor sizes were relatively larger in the LVI positive group, and there were more undifferentiated types. Overall, the pathologic stage was higher in the LVI positive group.

As seen in Supplemental Table 2, LVI positivity was more frequently observed as the stage increased, leading to a subgroup analysis. Before analyzing according to the TNM Stage, an initial analysis was conducted based on T stage, N stage, and LVI presence. In the T1 to T4 cohort, Group B is the N0 LVI positive group, which is essential for verifying the hypothesis that LVI positive has a prognosis similar to N1. Additionally, in the IB and IIA cohort, Group 2 is T2N0 LVI positive, which is significant because if LVI positive is interpreted as N1, the stage could shift from IB to IIA. In each cohort analysis, Groups B and 2 will be the primary focus for comparison with other groups.

### T1 cohort

A total of 3,187 patients with pathologic T1 were grouped into four categories based on N stage and LVI status, and their clinicopathologic characteristics were analyzed. Groups A and B were classified as stage IA, while groups C and D were classified as IB. Group A was younger, underwent less extensive LN dissection, and had smaller tumor sizes. Groups A and C (LVI negative) showed a higher proportion of undifferentiated type adenocarcinoma. No significant differences were observed among the groups when focusing on Group B (Supplemental Tables 3 − 1). In survival analysis, Group B showed statistically significant poorer overall survival (OS) and disease-specific survival (DSS) compared to Group A, with no significant difference when compared to Groups C and D. No differences in disease-free survival (DFS) were observed among all groups (Supplemental Fig. 1). In other words, in the T1 cohort, when N0 with LVI positive, the prognosis is poorer than N0 and similar to N1.

### T2 cohort

The same analysis was conducted on a total of 553 T2 patients. Groups A and B were in stage IB, while groups C and D were in stage IIA. Group A was younger and had smaller tumor sizes. No significant differences were observed in the extent of gastrectomy, LN dissection, histologic type, and number of harvested LNs (Supplemental Tables 3 − 2). In survival analysis, Group B showed statistically significant poorer OS and DSS compared to Group A. When compared to Groups C and D (stage IIA), Group B (stage IB, T2N0 LVI positive) showed no significant difference, but a trend towards poorer OS. Differences in DFS were observed between Groups A and D, with no differences between Group B and the other groups (Supplemental Fig. 2).

### T3 cohort

An identical analysis was executed on a cohort of 451 T3 patients. Groups A and B were categorized under stage IIA, while Groups C and D fell under stage IIB. Notably, Groups A and C consisted of younger patients. Other variables did not show significant differences across the groups (Supplemental Tables 3–3). In the survival analysis, Group B did not demonstrate significant disparities in OS, DSS, and DFS when compared to Groups A and C. Intriguingly, Group B exhibited superior OS, DSS, and DFS compared to Group D (Supplemental Fig. 3).

### T4 cohort

A parallel analysis was conducted on a cohort of 259 T4 patients. It’s essential to note that all patients within the T4 cohort were classified as T4a, with no instances of T4b. Groups A and B were designated under stage IIB, while Groups C and D were classified under stage IIIA. Some variations were observed in the surgical approach methods across the groups. However, these differences did not translate into significant disparities overall (Supplemental Tables 3–4). Across all groups, there were no discernible differences in OS, DSS, and DFS (Supplemental Fig. 4).

### IB and IIA cohort

**Fig. 1:**
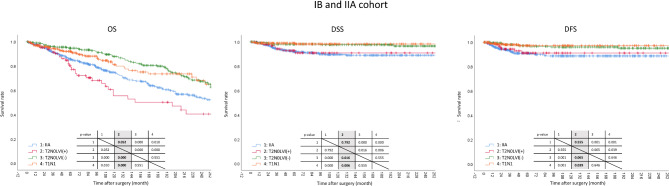
Survival analysis of IB and IIA cohort. Kaplan-Meier curves show Group 2 with significantly poorer OS compared to Groups 1, 3, and 4. Group 1 also demonstrates reduced OS in relation to Groups 3 and 4, though Groups 3 and 4 are similar in OS. In DSS, no significant differences are noted between Groups 1 and 2 or between Groups 3 and 4; however, both Groups 1 and 2 have markedly poorer DSS compared to Groups 3 and 4. DFS patterns are consistent with DSS findings, with a notable trend between Groups 2 and 3. This underscores varied prognostic outcomes based on group classifications

This cohort is pivotal for the validation of the study’s hypothesis. The analysis encompassed a total of 1,229 patients, with Group 1 falling under stage IIA and Groups 2, 3, and 4 categorized under stage IB. It was observed that Group 3 predominantly consisted of younger patients, whereas Group 2 had an older demographic. Some variations in gender distribution were noted across the groups. Group 3 exhibited a higher inclination towards open surgical approaches, and Group 1 had a more frequent occurrence of total gastrectomy. Group 4 had a pronounced proportion of D1 + LN dissection. Group 1 was characterized by larger tumor sizes, and there were subtle differences in histologic types across the groups (Table 1). In terms of survival analysis, Group 2 displayed a poorer OS compared to Groups 1, 3, and 4. Group 1 also had a diminished OS when compared to Groups 3 and 4, with no significant disparities observed between Groups 3 and 4. In DSS, Groups 1 and 2 did not show significant differences, and neither did Groups 3 and 4. However, Groups 1 and 2 were markedly poorer than Groups 3 and 4. DFS mirrored the results of DSS, with only a trend being observed between Groups 2 and 3 (Fig. 1).

**Table 1:** Clinicopathological characteristics of the IB and IIA cohort (*n* = 1,229)

	Group 1	Group 2	Group 3	Group 4	
Variables	IIA (*n* = 552)	T2N0 LVI(+) (IB) (*n* = 109)	T2N0 LVI(-) (IB) (*n* = 283)	T1N1 (IB) (*n* = 285)	*p*-value
Age (year)	59.3 ± 12.4	63.3 ± 12.2	58.2 ± 12.3	61 ± 12.1	0.001
Sex					0.005
Male	385 (69.7)	76 (69.7)	216 (76.3)	178 (62.5)	
Female	167 (30.3)	33 (30.3)	67 (23.7)	107 (37.5)	
BMI (kg/m2)	23.8 ± 3.1	23.7 ± 2.5	23.3 ± 3.2	23.7 ± 3.4	0.602
ECOG score					0.407
0 or 1	537 (97.3)	105 (96.3)	272 (96.1)	280 (98.2)	
2 or higher	15 (2.7)	4 (3.7)	11 (3.9)	5 (1.8)	
Approach					0.000
Open	426 (77.2)	74 (67.9)	234 (82.7)	156 (54.7)	
Laparoscopy	118 (21.4)	34 (31.2)	46 (16.3)	122 (42.8)	
Robot	8 (1.4)	1 (0.9)	3 (1.1)	7 (2.5)	
Extent of gastrectomy					0.001
Total gastrectomy	146 (26.4)	25 (22.9)	66 (23.3)	41 (14.4)	
Distal gastrectomy	404 (73.2)	83 (76.1)	215 (76)	244 (85.6)	
Proximal gastrectomy	2 (0.4)	1 (0.9)	2 (0.7)	0 (0)	
Extent of lymph node dissection					0.000
D1+	106 (19.2)	23 (21.1)	58 (20.5)	96 (33.7)	
D2 or more	446 (80.8)	86 (78.9)	225 (79.5)	189 (66.3)	
OP time	197.3 ± 52.7	183.7 ± 57.2	195.9 ± 45.6	186.3 ± 55.2	0.093
EBL	145 ± 109.5	124.1 ± 91.9	143.1 ± 122.3	139.8 ± 161.2	0.730
Number of tumor					0.228
Single	530 (96)	101 (92.7)	264 (93.3)	270 (94.7)	
Multiple	22 (4)	8 (7.3)	19 (6.7)	15 (5.3)	
Tumor size	4.7 ± 2.6	4.3 ± 2.3	3.7 ± 1.8	3.8 ± 2	0.000
Histology					0.008
Differentiated type	246 (44.6)	58 (53.2)	119 (42)	155 (54.4)	
Undifferentiated type	305 (55.4)	51 (46.8)	164 (58)	130 (45.6)	
Number of harvested lymph nodes	42.3 ± 17.2	40.9 ± 16.8	43.4 ± 18.1	40.4 ± 16	0.175

LVI, lymphovascular invasion; BMI, body mass index; ECOG, Eastern Cooperative Oncology Group; OP, operation; EBL, estimated blood loss

### Recurrence pattern

The recurrence patterns were analyzed in the IB and IIA cohorts. Additionally, post hoc analyses were conducted to examine whether recurrence patterns differed based on LVI status in T2 cases. Group 1 had a higher overall recurrence rate, with a significantly higher rate of recurrence in distant organs compared to Group 4. However, no significant differences were observed when compared to Group 2 or Group 3. Notably, Group 2 exhibited slightly higher recurrence rates in distant lymph nodes and distant organs compared to Groups 3 or 4, but the statistical significance was observed only compared to Group 4 (Table 2).

**Table 2:** Recurrence pattern

	Group 1	Group 2	Group 3	Group 4	
Variables	IIA (*n* = 552)	T2N0 LVI(+) (IB) (*n* = 109)	T2N0 LVI(-) (IB) (*n* = 283)	T1N1 (IB) (*n* = 285)	*p*-value
Recurrence pattern					0.008
No recurrence	505 (91.5)	102 (93.6)	273 (96.5)	279 (97.9)	
Locoregional recurrence	5 (0.9)	0 (0)	1 (0.4)	1 (0.4)	
Distant LNs	5 (0.9)	2 (1.8)	1 (0.4)	0 (0)	
Distant organs	37 (6.7)	5 (4.6)	8 (2.8)	5 (1.8)	
*p*-values of post hoc analyses					
vs. Group 1		0.493	0.051	0.002	
vs. Group 2	0.493		0.259	0.029	
vs. Group 3	0.051	0.259		0.669	
vs. Group 4	0.002	0.029	0.669		

LVI, lymphovascular invasion; LN, lymph node

### Risk factors for overall survival in T2BN0 patients

To determine whether LVI status independently impacts prognosis in T2N0 patients, a Cox regression analysis was performed for overall survival. Variables included in the analysis were age, sex, ECOG status, surgical approach, extent of gastrectomy, extent of lymph node dissection, tumor size, histological classification, and LVI status. The results indicated that both age and LVI positivity were independent prognostic factors, with a hazard ratio of 2.141 for LVI positivity (Table 3).

**Table 3:** Risk factors for overall survival in T2N0 patients

Variables	Univariate					Multivariate			
	HR	LCI	UCI	*p*-value		HR	LCI	UCI	*p*-value
Age	1.091	1.067	1.114	0.000		1.088	1.066	1.111	0.000
Sex									
Male	Reference								
Female	0.658	0.399	1.082	0.099		0.681	0.430	1.077	0.100
ECOG									
0 or 1	Reference								
2 or higher	0.573	0.138	2.374	0.442					
Approach									
Open	Reference								
Minimally invasive surgery	0.918	0.364	2.318	0.856					
Extent of gastrectomy									
Total gastrectomy	Reference								
Partial gastrectomy	1.003	0.578	1.740	0.992					
Extent of LN dissection									
D1+	Reference								
D2 or higher	1.144	0.616	2.126	0.670					
Tumor size	1.010	0.928	1.100	0.812					
Histological classification									
Differentiated type	Reference								
Undifferentiated type	1.078	0.712	1.632	0.722					
LVI status									
LVI negative	Reference								
LVI positive	2.117	1.372	3.267	0.001		2.141	1.398	3.280	0.000

HR, hazard ratio; LCI, lower confidence interval; UCI, upper confidence interval; ECOG, Eastern Cooperative Oncology Group; LN, lymph node; LVI, lymphovascular invasion

## Discussion

LN metastasis significantly influences the prognosis of gastric cancer. Consequently, gastric cancer treatment guidelines recommend harvesting at least 16 LNs to ensure accurate staging [2, 18]. The N stage in gastric cancer is determined by the number of metastatic LNs. However, diagnosing LN metastasis presents several challenges. Firstly, there is the issue of how many LNs hidden within fat-like tissue can be collected. These include the potential for LN omission during pathology review and the risk of false negatives depending on the LN slide section examined. Despite these challenges, such LN false negatives do not significantly impact prognosis. In gastric cancer, two main scenarios could lead to significant changes in treatment due to false negatives. The first scenario involves patients eligible for ESD. However, if LVI is determined to be positive after ESD, an additional radical gastrectomy is performed, which means the final outcome may not significantly differ [19]. The second scenario is related to stage migration, which determines the need for adjuvant chemotherapy. For example, while T2N0 might not indicate adjuvant chemotherapy as Stage IB, the presence of one LN metastasis changing the classification to T2N1 would make it Stage IIA, indicating the need for adjuvant chemotherapy. Therefore, false negatives in LN status in T2 patients can significantly influence the decision to administer adjuvant chemotherapy, thereby impacting prognosis [2]. 

There are various methods for predicting LN metastasis in gastric cancer. Similar to the criteria for ESD indications, factors such as depth of invasion, tumor size, and tumor histology can be utilized [6, 7, 20]. Additionally, LVI is a well-known risk factor for LN metastasis [21]. Lymphatics and vessels, located within the gastric submucosal layer, when found to contain tumor cells, are recognized as risk factors for LN metastasis [10]. Furthermore, studies have reported that LVI itself influences the prognosis of cancer, serving as an independent prognostic factor and part of the staging in various cancers such as colorectal cancer, breast cancer, urothelial carcinoma, endometrial cancer, and oral tongue squamous cell carcinoma [22–26]. However, in gastric cancer, while LVI is considered a high-risk factor for LN metastasis and is important for determining the curability of ESD, its significance beyond this context has not been as emphasized. Consequently, it has not yet been incorporated into the staging system [27–30]. 

Therefore, we analyzed the clinical significance of LVI by comparing the prognosis according to LVI status in node-negative patients. The patients were divided into cohorts based on their T stage, with the T2 cohort anticipated to show the most significant difference. The presence or absence of LN metastasis in the T2 cohort determines the indication for adjuvant chemotherapy [2, 31, 32]. Thus, a T2N0 patient with a false-negative node could be considered for adjuvant chemotherapy indication, but the actual stage being IB might result in not receiving chemotherapy. Indeed, the results of our study showed that the T2N0 group with positive LVI had a significantly poorer prognosis compared to the T2N0 group with negative LVI. There was no significant difference in prognosis between the T2N1 group regardless of LVI status. When comparing the T2N0 group with positive LVI to the T2N1 group, the T2N0 with positive LVI, classified as stage IB, exhibited a significantly poorer prognosis than the T2N1 group classified as stage IIA. This difference could be attributed to the variation in the administration of adjuvant chemotherapy.

In the T1 cohort, there was no significant difference based on LVI status in terms of long-term survival. This is likely because patients in this cohort were all classified as stage IA or IB, for which adjuvant chemotherapy is not indicated, and all had very good prognoses, resulting in no significant differences. Similarly, no significant differences in prognosis were observed in the T3 and T4 cohorts. For the T3 cohort, which could range from stage IIA to IIB, and the T4 cohort, which could range from stage IIB to IIIA, stage migration is possible. However, since all are indications for adjuvant chemotherapy, there was no difference in treatment modality, leading to no significant difference in prognosis.

Ultimately, to consider a revision of the stage for patients with T2N0 and positive LVI, a comparison was made with the stage IB and IIA cohorts. The results showed that T2N0 patients with positive LVI, belonging to stage IB, had a significant poor prognosis compared to those in the same stage IB, such as T1N1 and T2N0 with negative LVI, and no significant difference was observed with the stage IIA group. Additionally, T2N0 patients with positive LVI demonstrated a higher tendency for recurrence in distant LNs and distant organs compared to other stage IB patients. Moreover, LVI positivity was an independent risk factor for OS in T2N0 patients. These findings suggest that the T2N0 group with positive LVI could be considered for adjuvant chemotherapy as if they were in stage IIA.

This study has several limitations. Firstly, as a single-center retrospective study, there may be a selection bias. Additionally, to support the claims of this study, future prospective research is needed, which would involve administering adjuvant chemotherapy to the T2N0 group with positive LVI and directly comparing them with the stage IIA group. Despite these limitations, to the best of our knowledge, this study is the first to confirm the clinical significance of LVI in node negative T2 gastric cancer.

## Conclusion

This study highlights the clinical significance of LVI in node negative gastric cancer, suggesting LVI as a potential prognostic factor, particularly in the T2 cohort. The presence of LVI seemed to be associated with an increased risk of disease recurrence and poorer OS, reflecting a higher likelihood of occult LN involvement. These findings suggest that incorporating LVI into the staging system for node-negative gastric cancer could enhance prognostic accuracy and guide treatment decisions. Future research should aim to validate these observations and assess the potential benefits of staging modifications informed by LVI status.

## Electronic Supplementary Material

Below is the link to the electronic supplementary material.

Supplementary Material 1

Supplementary Material 2: Fig. 1. Survival analysis of T1 cohort. Kaplan-Meier curves depict Group 2 with significantly poorer OS and DSS compared to Group 1, and outcomes comparable to Groups 3 and 4. No significant differences in DFS were observed across all groups. This highlights the prognostic impact of LVI in node-negative patients.

Supplementary Material 3: Fig. 2. Survival analysis of T2 cohort. Kaplan-Meier curves show that Group 2 has significantly worse OS and DSS compared to Group 1. Outcomes for Group 2 are similar to Groups 3 and 4, with a trend towards poorer OS. DFS differs between Groups 1 and 4, while Group 2 shows comparable DFS to all other groups. This illustrates the nuanced impact of LVI on survival outcomes in early-stage gastric cancer.

Supplementary Material 4: Fig. 3. Survival analysis of T3 cohort. Kaplan-Meier curves illustrate that Group 2 shows no significant differences in OS, DSS, and DFS when compared to Groups 1 and 3. However, Group 2 exhibits significantly better OS, DSS, and DFS than Group 4. This suggests that factors specific to Group 2 may confer a survival advantage over those in Group 4.

Supplementary Material 5: Fig. 4. Survival analysis of T4 cohort. Kaplan-Meier curves display that there are no significant differences in OS, DSS, and DFS across all groups. This indicates a uniformity in survival outcomes irrespective of group distinctions within this cohort.

## Data Availability

No datasets were generated or analysed during the current study.

## Abbreviations

LVI: Lymphovascular invasion
OS: Overall survival
DSS: Disease-specific survival
DFS: Disease-free survival
LN: Lymph node
ESD: Endoscopic submucosal dissection
ECOG: Eastern Cooperative Oncology Group
