# Supplementary material for: Prognostic impact of lymphovascular invasion in node-negative gastric cancer: a retrospective cohort study
Source: World J Surg Oncol. 2024 Dec 20;22:340. doi: 10.1186/s12957-024-03629-6 (PMC11662831; doi:10.1186/s12957-024-03629-6)
Supplement: Supplementary file 1 — Supplementary Material 1 [file 12957_2024_3629_MOESM1_ESM.docx]

**Supplemental Table 1. Definition of the cohorts and groups**

| **Cohort** | **T1 (n = 3,187)** | **T2 (n = 553)** | **T3 (n = 451)** | **T4 (n = 259)** |
| --- | --- | --- | --- | --- |
| **Group A** | T1N0 LVI (-) | T2N0 LVI (-) | T3N0 LVI (-) | T4N0 LVI (-) |
| **Group B** | T1N0 LVI (+) | T2N0 LVI (+) | T3N0 LVI (+) | T4N0 LVI (+) |
| **Group C** | T1N1 LVI (-) | T2N1 LVI (-) | T3N1 LVI (-) | T4N1 LVI (-) |
| **Group D** | T1N1 LVI (+) | T2N1 LVI (+) | T3N1 LVI (+) | T4N1 LVI (+) |
|  |  |  |  |  |
| **Cohort** | **IB and IIA (n = 1,229)** | |  |  |
| **Group 1** | IIA |  |  |  |
| **Group 2** | T2N0 LVI (+) |  |  |  |
| **Group 3** | T2N0 LVI (-) |  |  |  |
| **Group 4** | T1N1 |  |  |  |

**Supplemental Table 2. Clinicopathological characteristics**

| Variables | LVI (-) (n = 3434) | LVI (+) (n = 2265) | p-value |
| --- | --- | --- | --- |
| Age (year) | 57.9 ± 12 | 60.2 ± 12.1 | 0.000 |
| Sex |  |  | 1.000 |
| Male | 2257 (65.7) | 1489 (65.7) |  |
| Female | 1177 (34.3) | 776 (34.3) |  |
| BMI (kg/m2) | 23.7 ± 3.1 | 23.6 ± 3.3 | 0.167 |
| ECOG score |  |  | 0.218 |
| 0 or 1 | 3316 (96.6) | 2201 (97.2) |  |
| 2 or higher | 118 (3.4) | 64 (2.8) |  |
| Approach |  |  | 0.000 |
| Open | 2017 (58.7) | 1792 (79.1) |  |
| Laparoscopy | 1319 (38.4) | 445 (19.6) |  |
| Robot | 98 (2.9) | 28 (1.2) |  |
| Extent of gastrectomy |  |  | 0.000 |
| Total gastrectomy | 660 (19.2) | 680 (30) |  |
| Distal gastrectomy | 2767 (80.6) | 1576 (69.6) |  |
| Proximal gastrectomy | 7 (0.2) | 9 (0.4) |  |
| Extent of lymph node dissection |  |  | 0.000 |
| D1+ | 1333 (38.8) | 401 (17.7) |  |
| D2 or more | 2101 (61.2) | 1864 (82.3) |  |
| OP time | 190.1 ± 50 | 193.2 ± 52.2 | 0.109 |
| EBL | 135 ± 144.3 | 156.5 ± 204.9 | 0.003 |
| Number of tumors |  |  | 0.010 |
| Single | 3246 (94.5) | 2175 (96) |  |
| Multiple | 188 (5.5) | 90 (4) |  |
| Tumor size | 3.1 ± 2.7 | 5.5 ± 3.1 | 0.000 |
| Histology |  |  | 0.000 |
| Differentiated type | 1658 (48.4) | 967 (42.7) |  |
| Undifferentiated type | 1768 (51.6) | 1297 (57.3) |  |
| Number of harvested lymph nodes | 41.1 ± 16.5 | 44.9 ± 17.3 | 0.000 |
| Pathologic T stage |  |  | 0.000 |
| T1 | 2737 (79.7) | 593 (26.2) |  |
| T2 | 338 (9.8) | 408 (18) |  |
| T3 | 240 (7) | 566 (25) |  |
| T4a | 112 (3.3) | 682 (30.1) |  |
| T4b | 7 (0.2) | 16 (0.7) |  |
| Pathologic N stage |  |  | 0.000 |
| N0 | 3196 (93.1) | 517 (22.8) |  |
| N1 | 172 (5) | 565 (24.9) |  |
| N2 | 48 (1.4) | 509 (22.5) |  |
| N3a | 15 (0.4) | 438 (19.3) |  |
| N3b | 3 (0.1) | 236 (10.4) |  |
| Stage (AJCC 8^th^ edition) |  |  | 0.000 |
| IA | 2626 (76.5) | 276 (12.2) |  |
| IB | 372 (10.8) | 305 (13.5) |  |
| IIA | 256 (7.5) | 294 (13) |  |
| IIB | 126 (3.7) | 317 (14) |  |
| IIIA | 44 (1.3) | 479 (21.1) |  |
| IIIB | 9 (0.3) | 381 (16.8) |  |
| IIIC | 1 (0) | 213 (9.4) |  |
| Follow up duration (mean) | 98.9 ± 80.7 | 80.8 ± 82.1 | 0.000 |
| Follow up duration (median [IQR]) | 72 [36-150] | 52 [21-108] | 0.000 |

Data given as numbers (%) and mean ± standard deviation.

**Supplemental Table 3-1. Clinicopathological characteristics of the T1 cohort (n = 3,187).**

|  | Group A | Group B | Group C | Group D |  |
| --- | --- | --- | --- | --- | --- |
| Variables | N0 LVI (-) (IA) (n = 2626) | N0 LVI (+) (IA) (n = 276) | N1 LVI (-) (IB) (n = 89) | N1 LVI (+) (IB) (n = 196) | p-value |
| Age (year) | 57.8 ± 11.9 | 63 ± 11.1 | 61.7 ± 10.8 | 60.7 ± 12.7 | 0.000 |
| Sex |  |  |  |  | 0.419 |
| Male | 1702 (64.8) | 169 (61.2) | 52 (58.4) | 126 (64.3) |  |
| Female | 924 (35.2) | 107 (38.8) | 37 (41.6) | 70 (35.7) |  |
| BMI (kg/m2) | 23.8 ± 3.1 | 24 ± 3 | 24.1 ± 3 | 23.5 ± 3.6 | 0.412 |
| ECOG score |  |  |  |  | 0.479 |
| 0 or 1 | 2537 (96.6) | 270 (97.8) | 88 (98.9) | 192 (98) |  |
| 2 or higher | 89 (3.4) | 6 (2.2) | 1 (1.1) | 4 (2) |  |
| Approach |  |  |  |  | 0.262 |
| Open | 1372 (52.2) | 127 (46) | 45 (50.6) | 111 (56.6) |  |
| Laparoscopy | 1163 (44.3) | 141 (51.1) | 43 (48.3) | 79 (40.3) |  |
| Robot | 91 (3.5) | 8 (2.9) | 1 (1.1) | 6 (3.1) |  |
| Extent of gastrectomy |  |  |  |  | 0.834 |
| Total gastrectomy | 452 (17.2) | 45 (16.3) | 12 (13.5) | 29 (14.8) |  |
| Distal gastrectomy | 2171 (82.7) | 231 (83.7) | 77 (86.5) | 167 (85.2) |  |
| Proximal gastrectomy | 3 (0.1) | 0 (0) | 0 (0) | 0 (0) |  |
| Extent of lymph node dissection |  |  |  |  | 0.003 |
| D1+ | 1167 (44.4) | 109 (39.5) | 31 (34.8) | 65 (33.2) |  |
| D2 or more | 1459 (55.6) | 167 (60.5) | 58 (65.2) | 131 (66.8) |  |
| OP time | 188.1 ± 48.8 | 179.2 ± 45.5 | 184.6 ± 66.4 | 187.3 ± 48.5 | 0.102 |
| EBL | 132.7 ± 148 | 125.5 ± 142.3 | 109.4 ± 88.5 | 156.3 ± 187.7 | 0.185 |
| Number of tumor |  |  |  |  | 0.642 |
| Single | 2472 (94.1) | 255 (92.4) | 84 (94.4) | 186 (94.9) |  |
| Multiple | 154 (5.9) | 21 (7.6) | 5 (5.6) | 10 (5.1) |  |
| Tumor size | 2.7 ± 2.6 | 3.3 ± 1.9 | 3.8 ± 2.1 | 3.8 ± 1.9 | 0.000 |
| Histology |  |  |  |  | 0.028 |
| Differentiated type | 1351 (51.6) | 166 (60.1) | 44 (49.4) | 111 (56.6) |  |
| Undifferentiated type | 1268 (48.4) | 110 (39.9) | 45 (50.6) | 85 (43.4) |  |
| Number of harvested lymph nodes | 40.6 ± 16.3 | 39.6 ± 16.1 | 41.2 ± 16.7 | 40 ± 15.7 | 0.777 |

LVI, lymphovascular invasion; BMI, body mass index; ECOG, Eastern Cooperative Oncology Group; OP, operation; EBL, estimated blood loss.

**Supplemental Table 3-2. Clinicopathological characteristics of the T2 cohort (n = 553).**

|  | Group A | Group B | Group C | Group D |  |
| --- | --- | --- | --- | --- | --- |
| Variables | N0 LVI (-) (IB) (n = 283) | N0 LVI (+) (IB) (n = 109) | N1 LVI (-) (IIA) (n = 37) | N1 LVI (+) (IIA) (n = 124) | p-value |
| Age (year) | 58.2 ± 12.3 | 63.3 ± 12.2 | 60.5 ± 9.1 | 60.5 ± 11.3 | 0.002 |
| Sex |  |  |  |  | 0.214 |
| Male | 216 (76.3) | 76 (69.7) | 32 (86.5) | 95 (76.6) |  |
| Female | 67 (23.7) | 33 (30.3) | 5 (13.5) | 29 (23.4) |  |
| BMI (kg/m2) | 23.3 ± 3.2 | 23.7 ± 2.5 | 23.7 ± 2.8 | 24.3 ± 3.1 | 0.281 |
| ECOG score |  |  |  |  | 0.101 |
| 0 or 1 | 272 (96.1) | 105 (96.3) | 33 (89.2) | 122 (98.4) |  |
| 2 or higher | 11 (3.9) | 4 (3.7) | 4 (10.8) | 2 (1.6) |  |
| Approach |  |  |  |  | 0.010 |
| Open | 234 (82.7) | 74 (67.9) | 27 (73) | 103 (83.1) |  |
| Laparoscopy | 46 (16.3) | 34 (31.2) | 9 (24.3) | 18 (14.5) |  |
| Robot | 3 (1.1) | 1 (0.9) | 1 (2.7) | 3 (2.4) |  |
| Extent of gastrectomy |  |  |  |  | 0.976 |
| Total gastrectomy | 66 (23.3) | 25 (22.9) | 7 (18.9) | 29 (23.4) |  |
| Distal gastrectomy | 215 (76) | 83 (76.1) | 30 (81.1) | 95 (76.6) |  |
| Proximal gastrectomy | 2 (0.7) | 1 (0.9) | 0 (0) | 0 (0) |  |
| Extent of lymph node dissection |  |  |  |  | 0.966 |
| D1+ | 58 (20.5) | 23 (21.1) | 7 (18.9) | 23 (18.5) |  |
| D2 or more | 225 (79.5) | 86 (78.9) | 30 (81.1) | 101 (81.5) |  |
| OP time | 195.9 ± 45.6 | 183.7 ± 57.2 | 188.4 ± 45.2 | 200.6 ± 54.5 | 0.322 |
| EBL | 143.1 ± 122.3 | 124.1 ± 91.9 | 102.9 ± 77 | 181.2 ± 139.7 | 0.026 |
| Number of tumor |  |  |  |  | 0.895 |
| Single | 264 (93.3) | 101 (92.7) | 35 (94.6) | 118 (95.2) |  |
| Multiple | 19 (6.7) | 8 (7.3) | 2 (5.4) | 6 (4.8) |  |
| Tumor size | 3.7 ± 1.8 | 4.3 ± 2.3 | 4.4 ± 2.4 | 4.5 ± 2.2 | 0.001 |
| Histology |  |  |  |  | 0.085 |
| Differentiated type | 119 (42) | 58 (53.2) | 16 (43.2) | 66 (53.2) |  |
| Undifferentiated type | 164 (58) | 51 (46.8) | 21 (56.8) | 58 (46.8) |  |
| Number of harvested lymph nodes | 43.4 ± 18.1 | 40.9 ± 16.8 | 41.3 ± 14.5 | 43.2 ± 16.9 | 0.571 |

LVI, lymphovascular invasion; BMI, body mass index; ECOG, Eastern Cooperative Oncology Group; OP, operation; EBL, estimated blood loss.

**Supplemental Table 3-3. Clinicopathological characteristics of the T3 cohort (n = 451).**

|  | Group A | Group B | Group C | Group D |  |
| --- | --- | --- | --- | --- | --- |
| Variables | N0 LVI (-) (IIA) (n = 202) | N0 LVI (+) (IIA) (n = 81) | N1 LVI (-) (IIB) (n = 26) | N1 LVI (+) (IIB) (n = 142) | p-value |
| Age (year) | 57.9 ± 12.7 | 62.6 ± 12 | 57.3 ± 14.2 | 61.9 ± 11.8 | 0.003 |
| Sex |  |  |  |  | 0.874 |
| Male | 135 (66.8) | 56 (69.1) | 18 (69.2) | 101 (71.1) |  |
| Female | 67 (33.2) | 25 (30.9) | 8 (30.8) | 41 (28.9) |  |
| BMI (kg/m2) | 23.5 ± 2.7 | 23.2 ± 4 | 23.7 ± 2.6 | 23.1 ± 3.1 | 0.827 |
| ECOG score |  |  |  |  | 0.960 |
| 0 or 1 | 196 (97) | 79 (97.5) | 26 (100) | 139 (97.9) |  |
| 2 or higher | 6 (3) | 2 (2.5) | 0 (0) | 3 (2.1) |  |
| Approach |  |  |  |  | 0.537 |
| Open | 170 (84.2) | 65 (80.2) | 24 (92.3) | 125 (88) |  |
| Laparoscopy | 30 (14.9) | 15 (18.5) | 2 (7.7) | 17 (12) |  |
| Robot | 2 (1) | 1 (1.2) | 0 (0) | 0 (0) |  |
| Extent of gastrectomy |  |  |  |  | 0.531 |
| Total gastrectomy | 72 (35.6) | 24 (29.6) | 5 (19.2) | 50 (35.2) |  |
| Distal gastrectomy | 129 (63.9) | 56 (69.1) | 21 (80.8) | 91 (64.1) |  |
| Proximal gastrectomy | 1 (0.5) | 1 (1.2) | 0 (0) | 1 (0.7) |  |
| Extent of lymph node dissection |  |  |  |  | 0.901 |
| D1+ | 37 (18.3) | 15 (18.5) | 6 (23.1) | 25 (17.6) |  |
| D2 or more | 165 (81.7) | 66 (81.5) | 20 (76.9) | 117 (82.4) |  |
| OP time | 209.9 ± 52.3 | 192 ± 56.2 | 220.8 ± 50.6 | 198.4 ± 48.2 | 0.178 |
| EBL | 164.2 ± 100.7 | 128.4 ± 108.1 | 170 ± 78.5 | 130.5 ± 96 | 0.139 |
| Number of tumor |  |  |  |  | 0.355 |
| Single | 197 (97.5) | 76 (93.8) | 26 (100) | 136 (95.8) |  |
| Multiple | 5 (2.5) | 5 (6.2) | 0 (0) | 6 (4.2) |  |
| Tumor size | 4.8 ± 2.8 | 5.4 ± 2.6 | 4.8 ± 2.3 | 5.3 ± 2.3 | 0.173 |
| Histology |  |  |  |  | 0.057 |
| Differentiated type | 74 (36.8) | 36 (44.4) | 10 (38.5) | 73 (51.4) |  |
| Undifferentiated type | 127 (63.2) | 45 (55.6) | 16 (61.5) | 69 (48.6) |  |
| Number of harvested lymph nodes | 42.2 ± 17.1 | 43.1 ± 20.6 | 44.8 ± 15.1 | 43.8 ± 15.2 | 0.795 |

LVI, lymphovascular invasion; BMI, body mass index; ECOG, Eastern Cooperative Oncology Group; OP, operation; EBL, estimated blood loss.

**Supplemental Table 3-4. Clinicopathological characteristics of the T4 cohort (n = 259).**

|  | Group A | Group B | Group C | Group D |  |
| --- | --- | --- | --- | --- | --- |
| Variables | N0 LVI (-) (IIB) (n = 85) | N0 LVI (+) (IIB) (n = 51) | N1 LVI (-) (IIIA) (n = 20) | N1 LVI (+) (IIIA) (n = 103) | p-value |
| Age (year) | 56.2 ± 12.5 | 60.6 ± 13.2 | 55.3 ± 14.3 | 59.5 ± 13.9 | 0.139 |
| Sex |  |  |  |  | 0.258 |
| Male | 47 (55.3) | 35 (68.6) | 9 (45) | 59 (57.3) |  |
| Female | 38 (44.7) | 16 (31.4) | 11 (55) | 44 (42.7) |  |
| BMI (kg/m2) | 23.2 ± 3.2 | 23.2 ± 2.9 | 21.7 ± 2.7 | 23.6 ± 3.7 | 0.283 |
| ECOG score |  |  |  |  | 0.812 |
| 0 or 1 | 82 (96.5) | 50 (98) | 20 (100) | 98 (95.1) |  |
| 2 or higher | 3 (3.5) | 1 (2) | 0 (0) | 5 (4.9) |  |
| Approach |  |  |  |  | 0.013 |
| Open | 78 (91.8) | 43 (84.3) | 14 (70) | 91 (88.3) |  |
| Laparoscopy | 7 (8.2) | 5 (9.8) | 6 (30) | 12 (11.7) |  |
| Robot | 0 (0) | 3 (5.9) | 0 (0) | 0 (0) |  |
| Extent of gastrectomy |  |  |  |  | 0.457 |
| Total gastrectomy | 29 (34.1) | 20 (39.2) | 10 (50) | 46 (44.7) |  |
| Distal gastrectomy | 56 (65.9) | 30 (58.8) | 10 (50) | 56 (54.4) |  |
| Proximal gastrectomy | 0 (0) | 1 (2) | 0 (0) | 1 (1) |  |
| Extent of lymph node dissection |  |  |  |  | 0.441 |
| D1+ | 16 (18.8) | 7 (13.7) | 2 (10) | 11 (10.7) |  |
| D2 or more | 69 (81.2) | 44 (86.3) | 18 (90) | 92 (89.3) |  |
| OP time | 214.1 ± 66 | 211 ± 42.2 | 200.9 ± 52.3 | 204 ± 45.4 | 0.814 |
| EBL | 171.2 ± 119 | 97.7 ± 61.7 | 174 ± 113.5 | 147.4 ± 127.4 | 0.107 |
| Number of tumor |  |  |  |  | 0.151 |
| Single | 85 (100) | 49 (96.1) | 20 (100) | 98 (95.1) |  |
| Multiple | 0 (0) | 2 (3.9) | 0 (0) | 5 (4.9) |  |
| Tumor size | 6 ± 3.3 | 7 ± 3.5 | 7.2 ± 3.7 | 7.2 ± 3.5 | 0.100 |
| Histology |  |  |  |  | 0.390 |
| Differentiated type | 19 (22.4) | 15 (29.4) | 2 (10) | 23 (22.3) |  |
| Undifferentiated type | 66 (77.6) | 36 (70.6) | 18 (90) | 80 (77.7) |  |
| Number of harvested lymph nodes | 44.5 ± 16.6 | 43.5 ± 15.7 | 48.4 ± 20.6 | 45.4 ± 15.1 | 0.706 |

LVI, lymphovascular invasion; BMI, body mass index; ECOG, Eastern Cooperative Oncology Group; OP, operation; EBL, estimated blood loss.
